# COVID-19 in Children: Respiratory Involvement and Some Differences With the Adults

**DOI:** 10.3389/fped.2021.622240

**Published:** 2021-03-29

**Authors:** Jenny Libeth Jurado Hernández, Iván Francisco Álvarez Orozco

**Affiliations:** ^1^Department of Pediatric Pulmonology, Fundación Neumológica Colombiana, Bogotá, Colombia; ^2^Department of Pediatric Pulmonology, Neumocenter, Valledupar, Colombia

**Keywords:** SARS-CoV-2, COVID-19, respiratory system, respiratory involvement, pneumonia, ARDS, children

## Abstract

The coronavirus disease 2019 (COVID-19) represents a health problem with multidimensional impacts and heterogeneous respiratory involvement in children, probably due to the interaction between different and complex mechanisms that could explain its variable degrees of severity. Although the majority of reports reveal that children develop less severe cases, the number of patients is increasing with more morbidity. Most serious respiratory manifestations are acute respiratory distress syndrome (ARDS) and pneumonia. By understanding the key aspects that can be used to differentiate between pediatric and adult respiratory compromise by COVID-19, we can improve our knowledge, and thus decrease the negative impact of the disease in the pediatric population. In this mini review, we summarize some of the mechanisms and findings that distinguish between adult and pediatric COVID-19 and respiratory involvement, taking into account some issues related to the physiopathology, diagnosis, clinical and paraclinical presentation, severity, treatment, and control of the disease.

## Introduction

The coronavirus disease 2019 (COVID-19) is the result of infection by severe acute respiratory syndrome coronavirus 2 (SARS-CoV-2) (1, 2). This coronavirus is characterized by its high level of transmissibility and pathogenicity, resulting in a pandemic, and its multidimensional impact (3–5). SARS-CoV-2 produces heterogeneous respiratory involvement, especially in children (6, 7). Some vascular, immunological, and molecular mechanisms probably explain its variable degrees of severity or atypical presentations compared to adults (8) and, consequently, some differences in diagnosis, severity, treatment, and control of the disease. Currently, pediatric patients with severe manifestations of the disease are increasing (9); pneumonia is the most common respiratory entity, and acute respiratory distress syndrome (ARDS) is the critical form (7).

With the aim of summarizing the mechanisms and findings that can be used to differentiate respiratory involvement between pediatric and adult COVID-19, taking into account issues related to the physiopathology, diagnosis, severity, treatment, and control of the disease, this paper elaborates on the characteristics of the SARS-CoV-2 pathophysiology, clinical and paraclinical presentation, diagnostic and therapeutic approach, and follow-up of COVID-19 in both populations. Using the Scopus and PubMed databases, the keywords SARS-CoV-2, COVID-19, respiratory involvement, respiratory system, pneumonia, and ARDS were searched. This review includes the differences in COVID-19 manifestations between children and adults.

## Epidemiological Issues

COVID-19 occurs in children of all ages (10); however, the pediatric disease represents <5% of total cases (11). In this population, the infection predominates in school children and adolescents (12); the Center of Disease Control and Prevention (CDC) reports 386,329 cases in the United States (13). The percentage of cases is slightly higher in females compared with males (50.5 vs. 49.5%), and the rate of mortality is extremely low in both populations (<0.1%; 62 deaths) with a greater percentage of deaths in males (52.5%) (14). Despite these statistics, global data about morbidity in children may be understated because they have less frequent exposure to some sources of transmission, and the clinical course includes milder respiratory symptoms compared with adults (15–18). These situations may explain why children are less often tested (18, 19). For them, transmission of infection through familial clusters predominates (10).

## Pathophysiology

**Figure 1:**
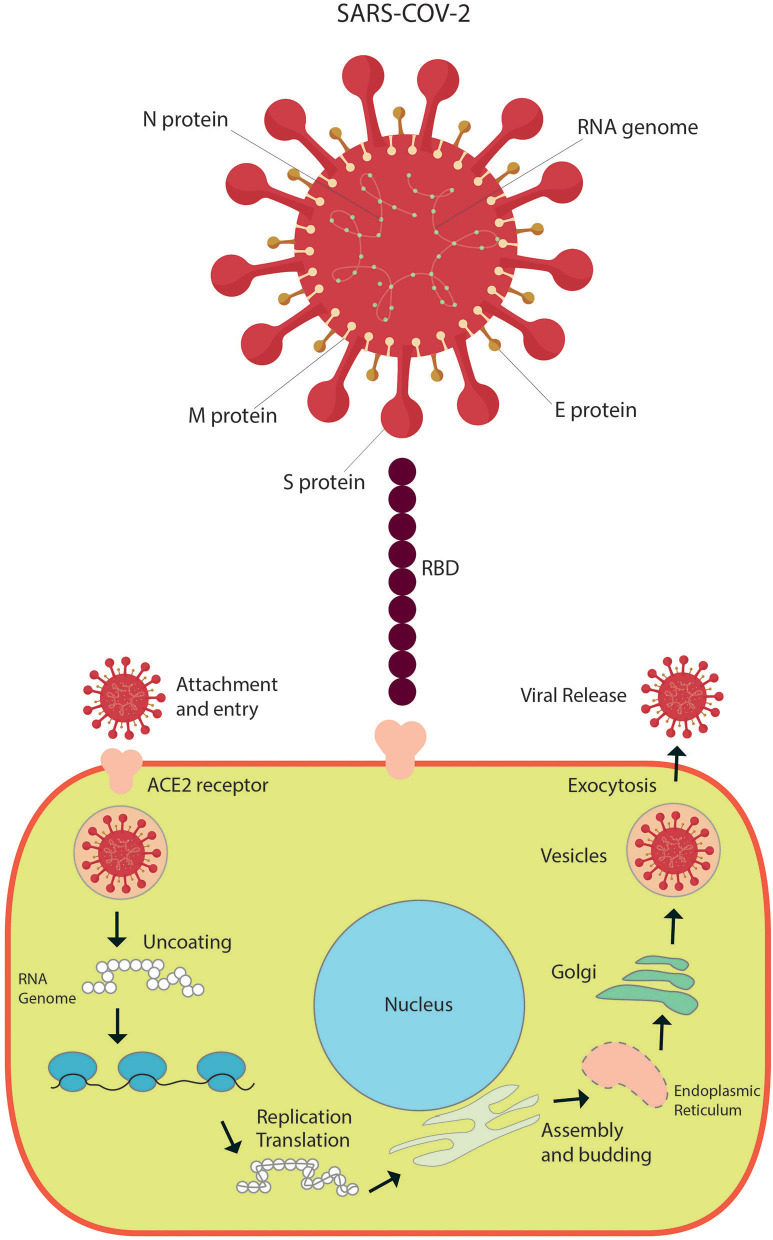
Basic structure of SARS-CoV-2 and pathophysiology of infection.

The Figure 1 shows the basic structure of SARS-CoV-2 and pathophysiology of COVID-19. SARS-CoV-2 infection causes heterogeneous respiratory involvement ranging from mild to severe respiratory failure. In pediatric and adult patients, this compromise can occur in three phases. In the first phase, the virus binds to epithelial cells of the respiratory tract to commence primary replication; most patients are able to contain the infection in this stage and thus present mild disease. In the second phase, SARS-CoV-2 migrates down the airways and enters alveolar epithelial cells facilitating pulmonary viral replication and localized inflammation (7, 20); most patients need hospitalization due to pneumonia. In the third phase, the rapid replicative process of the virus at the lung level may trigger apoptosis of cells with vascular leakage and the release of pro-inflammatory proteins (5). The simultaneous downregulation of ACE2 expression can alter the renin-angiotensin system with elevation of angiotensin-2, which increases inflammation and vascular permeability, causing pulmonary edema. Patients can develop a strong immune response (21, 22) with subsequent cytokine storm [e.g., release of IL-2, IL-6, IL-7, IL-10, GCSF, IP-10, MCP-1, MIP-1, and TNF-α; (5)] which causes ARDS and respiratory failure (23–25). The proportion of T cells (helper T cells and memory helper T cells) is diminished, and naïve helper T cell levels are elevated in the severe disease (5, 14). To date, pediatric moderate and critical respiratory cases are less frequent than those presented in adults.

Generally, the SARS-CoV-2 viral load is elevated in the first week of clinical presentation, gradually reducing afterward (26). However, after 4–7 days of COVID-19, some patients present a critical evolution concurrent with a decrease in viral load and deterioration of inflammatory parameters. Among the more severe clinical cases, some patients can have a less steep and prolonged decline in the SARS-CoV-2 load (5). Around days 7–10 of symptoms, an elevation in IgG and IgM levels against antigens of the virus appears, and there is a progressive decrease in the viral load (5, 27, 28). The persistence of high viral load and exaggerated inflammatory response in severe lung involvement and multi-organ dysfunction is explained by the combination of virus-mediated cytopathic effects and immunologically mediated injury. Patients can gradually improve, or they do not recover (29).

Mechanisms have been proposed to explain the lower severity of respiratory involvement by COVID-19 in children compared to adults (30). From an immunological standpoint, the function of innate immunity with a predominance of natural killer cells and the previous development of immunological memory against other respiratory infectious processes seem to influence the response capacity against this beta coronavirus, minimizing its clinical impact (5, 31, 32). Likewise, the availability of a greater number of B and T lymphocytes observed in children can prevent an excessive inflammatory response, conferring a less severe course of illness (33), especially in pneumonia (34). In this way, another potential route that can provide clinical benefit is constant immunological training secondary to frequent exposure to early childhood vaccines, including immunization against HiB and pneumococcus (35).

At the vascular level, endothelial function, and coagulation are more preserved in children, reducing the possibility of vasculitic alterations or pulmonary thrombotic phenomena (36).

From a microbiological context, the presence of additional viral infections concomitant to SARS-CoV-2 occurs more frequently in children than in adults and seems to play a protective role by inhibiting the replicative process (37–40). Furthermore, the competitive effect of normal airway microbiota can decrease colonization and growth of the virus (41, 42) and interfere with the appearance or infection severity. Previous infection with other coronaviruses may influence development of cross-protection against the novel coronavirus (43).

In children, exposure to SARS-CoV-2 is usually less frequent and has a lower intensity due to adopted precautionary measures, favoring a milder clinical presentation of COVID-19 (44).

From a molecular perspective, in the pediatric and adolescent population with COVID-19, increased ACE2 activity is observed. This characteristic appears to have a protective function due to its participation in anti-inflammatory signaling (43, 45), leading to less severe disease in children compared to that in the elderly (29). However, Sharif-Askari et al. reported a low expression level of TMPRSS2 and ACE2 in the upper and the lower respiratory tract of children and adolescents in comparison to adults with COPD or who smoked, suggesting the negative impact of some clinical conditions on the severity of COVID-19 in adults (46).

## Diagnosis

The demonstration of SARS-CoV-2 through the reverse transcription polymerase chain reaction (RT-PCR) test confirms diagnosis (15, 19, 28, 47); it is particularly useful in children given their heterogeneity in presentation of COVID-19 (12). In some cases, it is possible to find patients with a high index of suspicion for this viral infection with suggestive symptoms and/or a history of exposure and a negative test result in whom it can be necessary to repeat (48).

Moreover, the serology is available to recognize the population with prior or recent SARS-CoV-2 infection (6). However, this test is not considered the only tool to support diagnosis of acute infection because of the variability in the time for the seroconversion or the detection of antibodies against SARS-CoV-2 (6, 43). The serological assay may be helpful in the case of an individual with a high likelihood of infection whose molecular diagnosis and antigen test show false-negative results (6, 49, 50). Unlike adults, the seroconversion rate can be lower among infected children (51, 52), which suggests a weaker seroconversion in asymptomatic cases and slight forms of COVID-19 (43). Rostad et al. found IgG antibodies to the virus in all children with multisystem inflammatory syndrome in children (MIS-C) and in more than 90% of those with severe or moderate COVID-19 by clinic and inflammatory parameters; however, antibody responses in children with a mild form were not detected (53). With these findings, a prognostic and diagnostic role of antibodies specifically for pediatric COVID-19 is suggested.

## Clinical Presentation

In about two thirds of pediatric cases of COVID-19, the child had physical contact with a confirmed case (54), and the exposure, different from adults, usually occurred at home (55). In children, most described symptoms are fever (47.5–51.6%) and cough (41.5–47.3%) (7, 10, 18, 21, 22, 36, 56, 57); however, dyspnea (40%) is the most common respiratory signal in more severe presentations, such as pneumonia and ARDS (7, 18). Relative to adults, pediatric cases present other concomitant symptomatology (e.g., fatigue and muscle pain) and more co-morbid conditions even in different systems in a representative proportion (55, 58).

The progression to severe or critical forms is infrequent in pediatric cases (10): 2% are severe cases, and <2% correspond to critical evolutions (7). Some cases are classified as moderate disease due to radiological findings, although the symptoms are few (2, 7, 59). The recovery is faster probably due to lower affectation and a better immune response (60), but complications in the presence of co-morbidities are more likely (10, 61, 62). Although mortality in cases requiring pediatric intensive care units (PICU) is low (12, 63), an increase is being observed.

Götzinger et al. explored various risk factors for admission to intensive care. Age below 1 month, male sex, clinical evolution with lower airway infectious compromise, and a history of co-morbidities showed relevance. They also identified a heterogeneity of previous diseases, including pulmonary entities, cardiac disturbances, malignant diseases, or nervous system disorders. Some patients received antivirals or immunomodulators due to a serious clinical course (64); however, the role of these agents in pediatric COVID-19 is not fully established.

## Radiological Findings

The American College of Radiology suggests performing chest x-rays (CXR) in pediatric patients with moderate or severe symptomatology, and in those with antecedents and previous risk factors (18) because they can need hospitalization and greater care (65). Peribronchial cuffing in both lung fields and central and peripheral ground-glass opacities (GGOs) are present in this group. However, these patterns are still non-specific. Another finding is bilateral or unilateral consolidation. Less common presentations include pleural effusion and mediastinal widening. During follow-up, radiological control depends on clinical evolution with rapid resolution of involvement in most of the cases. If patients worsen, a persistence of symptom exacerbation in findings or new consolidations can be observed (66).

Palabiyik et al. observed alterations in CXR in about half of children evaluated for pneumonia, particularly in the lower areas. The most frequent abnormality was unilateral increased density (67). Unlike adults, radiological compromise is less described in children possibly because the cases are mostly mild, the disease goes unnoticed, or it is poorly evaluated (68). Some authors emphasize atypical manifestations in pediatric pneumonia, including unilateral lobar or segmental consolidation, central bilateral or unilateral GGOs, and/or consolidation, single-round consolidation, pleural effusion, or lymphadenopathy (65, 69).

The COVID-19 alterations most recognized on a chest computerized tomography (CT) scan in pediatrics are GGOs and patchy shadowing (7). Although, this imaging study is not recommended for systematic use, it has shown utility mainly in the evaluation of children when the acute clinical course includes hypoxemia or dyspnea, deterioration in clinical or laboratory parameters (for example, a higher D-dimer), or there is a poor response to support therapy (9, 65, 66). Unlike adults, CT indications are more specific, such as in clinical worsening or suspicion of pulmonary embolism (30, 65), because the avoidance of radiation is necessary. Typical features more reported in pneumonia are bilateral, peripheral, and/or sub-pleural GGOs and/or consolidation, especially in the lower lobe—and the “halo” sign. In relation to the indeterminate pattern, the CT scan reveals similar findings to those previously described in CXR and in “crazy paving” signs. Discrete small nodules (tree-in-bud, centrilobular) and lung cavitation can be observed in atypical presentations as well as other alterations mentioned in the radiography. In cases of indeterminate or atypical patterns, it is recommended that additional investigations of differential diagnoses occur according to each case (61, 69).

In comparison to adults, children have a generalized peripheral distribution of lesions (68) and a lower percentage of cases with GGOs, consolidation, crazy paving pattern, pleural effusion, and bilateral compromise (70). The more pronounced difference between the groups corresponds to a higher frequency of unilateral lesions (30% of cases) and nodules (15%) in children (70, 71); this implies that atypical presentations should be considered more often in pediatrics (67). Also, ~20% of the pediatric population have normal CT, which reinforces the importance of its performance in selected cases (70).

## Laboratory Data

Most children with COVID-19 have normal laboratory findings compared with adults; however, a variability in features is recognized (18). In a review that included 655 pediatric patients, 17.1% showed low leucocyte levels, and 13.3% had lymphopenia or neutropenia (18). In other publications that summarize various studies, high levels of ferritin in 26% of children (12), elevation of C-reactive protein in 19%, and procalcitonin in 25–31% have been reported (5, 12). All these alterations present possibly in the context of greater severity secondary to a more inflammatory response. In contrast, a lower presence of marked inflammatory changes and lymphopenia is possible (72). In relation to co-infection, the prevalence of other common respiratory pathogens is high in children; therefore, concomitant evaluation (37) in the peak season for viral respiratory illness is suggested (73).

In a study of 70 adolescents admitted to PICUs, 21 (30%) developed ARDS even in the first 2 weeks of admission with a prolonged hospital stay; most patients had bilateral infiltrates. The platelet counts were significantly lower, and levels of IL-6 were more elevated compared to those without ARDS. Although other markers (lactate, pro-B-type natriuretic peptide) were high, the results did not have statistical relevance (74). The overall mortality was 2.8% (74); however, in adults the percentage of mortality with this condition is higher.

## Treatment

Generally, children with severe and critical presentations of COVID-19 require hospitalization. In addition, patients with non-severe forms and risk of severe disease due to pre-existing conditions can need hospital admission. Pediatric treatment focuses on supportive care by respiratory support with supplemental oxygen and invasive or non-invasive ventilation, fluid and electrolyte support, judicious use of empiric antibiotics as indicated for community-acquired or healthcare-associated pneumonia, systematic clinical follow-up, and laboratory monitoring (75). Unlike adults, anticoagulation seems to be less frequent in children, shown by lower presentation of embolic events. Evaluation of inflammation with C-reactive protein, D-dimer, LDH, ferritin, and IL-6 can be considered two to three times per week or if there is clinical worsening (75, 76). Other laboratory or imaging studies can be required according to each case. Special attention must be given to adequate nutritional support and temperature control. Table 1 summarizes respiratory management in children (49, 77).

**Table 1:** Key points in respiratory management of Covid-19 in Children (49, 77).

**Oxygen therapy** (according to patient's evolution). • Low flow system: mild hipoxemia. • High flow system: moderate to severe hipoxemia, with precautions to reduce risk of dispersing contaminating aerosols.
**Invasive mechanical ventilation** (if respiratory failure, or persistent hipoxemia (Pa02/Fi02 < 200 or Sa/Fi < 264), increased need for oxygen or worsening tachypnea in patient on high-flow nasal cannula). • Protective mechanical ventilation. • Initial parameters: ✓ Tidal volume (TV): 4–8 ml/ kg, with monitoring of plateau pressure (if > 30 cm H20, decrease TV). ✓ Respiratory rate: 22–30 bpm (patients from 1 month to 2 years), 18–24 bpm (2–4 years), 14–20 bpm (patients > 8 years). ✓ Inspiration/expiration ratio (I: E ratio):1-2. ✓ End-expiratory pressure (PEEP): titrate according to oxygenation, arterial gases and CXR. Each increase of 2 cm H_2_0 as required. • Fraction of inspired O_2_ (FiO_2_): start at 100% and rapidly reduce to less than 60% in the first 2–6 h. ✓ Alarms: 10% above and below the parameters. Driving pressure < 15 cm H_2_0 and Plateau pressure < 30 cm H_2_0.
**Prone position** (in patients with moderate to severe ARDS who need oxygen therapy or mechanical ventilation).
**Glucocorticoids** (as adjunctive therapy in select cases). • Prednisolone 1 mg/kg orally or NG once daily (maximum dose 40 mg). • Dexamethasone 0.15 mg/kg orally, intravenously (IV), or nasogastrically once daily (maximum dose 6 mg). • Methylprednisolone 0.8 mg/kg IV once daily (maximum dose 32 mg). • Hydrocortisone: for patients ≥1 month: 1.3 mg/kg IV every 8 hours (maximum dose 50 mg; maximum total daily dose 150 mg). For neonates: 0.5 mg/kg IV every 12 h for 7 days followed by 0.5 mg/kg IV once daily for 3 days.
**Clinic follow-up** • Evaluation of vital signs, symptoms, and signals of respiratory worsening. According to findings, it is recommended the adjustment to care plan. • Judicious use of empiric antibiotic as indicated for community-acquired or health care-associated pneumonia. • Special attention to patients with chronic respiratory disease (severe asthma, cystic fibrosis, bronchopulmonary dysplasia, tracheostomy) or other complex uncontrolled conditions due to risk of an inadequate evolution.

There is no specific treatment for adult and pediatric COVID-19, and pharmacotherapy is controversial (64, 78). Therefore, clinical trials have been developed to investigate the use of antivirals, antimalarials, and adjunct therapies especially in adult patients with severe or non-severe manifestations. Other investigations have evaluated the impact of using different forms of oxygen therapy and prevention measures (79, 80). Few studies have included children. The main limitation in the research on antiviral therapy and other strategies has been the big difference in the number of pediatric and adult events in COVID-19 and asymptomatic infection or with minimal symptoms among children (81).

There are some conditional suggestions for the utilization of antiviral agents in pediatric COVID-19 given the lack of demonstrated efficacy. This therapy should be reserved for children with severe involvement secondary to confirmed disease. Remdesivir, a nucleotide analog prodrug that inhibits viral RNA polymerases (82), has been considered in pediatrics because randomized trials in adults suggest a potential benefit; however, the use of this agent must be individualized and, preferably, in the context of a clinical trial (75, 77, 81, 83). At this time, remdesivir is approved by the FDA for the treatment of COVID-19 in hospitalized patients aged >12 years and >40 kg in weight (84). Lopinavir/ritonavir is not recommended for routine use in children due to unfavorable pharmacodynamics and the absence of evidence for efficacy (77, 85, 86). Hydroxychloroquine and chloroquine are considered only in clinical trials and in hospitalization (77, 87–90).

As part of adjunctive therapy, low-dose glucocorticoids are suggested for select children with severe or critical disease who cannot participate in a clinical trial: the efficacy is uncertain given children have been underrepresented in the clinical trials (75, 79–81, 90–93). The use of other adjunctive treatment must be discussed case by case according to disease severity and in agreement with multidisciplinary teams as indicated (90). IL-6 inhibitors, interferon-beta 1b, and convalescent plasma from recovered COVID-19 patients are not recommended for routine use because the benefits/risks are uncertain in children (83, 84, 90, 94). Moreover, the clinical potential of other immunomodulators or passive immunization therapies must be elucidated with prospective, randomized, placebo-controlled trials in the pediatric group. Recently, evidence of potential therapeutic options in COVID-19 has been updated; however, it is necessary to develop high-quality trials to improve disease management (95).

Preventive measures to reduce viral spread utilize personal hygiene maintenance, including frequent hand washing, use of a face mask, disinfection of surfaces, social and physical distancing, home isolation, voluntary home quarantine, and operation adjustments in educative centers (77, 96).

Vaccination seems be the most effective method to avoid and control the illness (97, 98). Strategies include recombinant vectors, DNA, mRNA in lipid nanoparticles, protein subunits, inactivated viruses, and live attenuated viruses (7, 97, 99). Recently, the FDA approved the emergency use of two vaccines to prevent SARS-CoV-2 infection in individuals >16 years, generating profound worldwide expectation (100).

## Conclusions

Various mechanisms and findings can be used to differentiate between adult and pediatric COVID-19, especially with respiratory involvement; however, children are less often tested. They have lower seroconversion and less exposure to some sources of transmission, although infection through familial clusters predominates. Adaptive and innate immune responses, previous or concomitant infection with other viruses, microbiota effects, increased ACE2 activity, and more preserved coagulation and endothelial function confer clinical advantages that contribute to the presentation of milder forms of the disease and a better prognosis. Fever and cough are more common manifestations, and dyspnea occurs in the context of pneumonia and ARDS. Co-morbidities can affect evolution. Most children show normal laboratory findings; however, there is certain variability and a lower prevalence of lymphopenia or marked inflammatory parameters. They can present atypical or normal images in a representative proportion of cases. CXR is preferred over CT. A CT scan is performed if there is clinical worsening or suspicion of pulmonary embolism. Pediatric treatment focuses on supportive care as there is less research into vaccines and specific treatments and thus a more conditioned use of pharmacotherapy.

Although there are still knowledge gaps in pediatric COVID-19 discussion, it is necessary to continue comprehensive and specific investigations to mitigate its consequences.

## Author Contributions

All authors listed have made a substantial, direct and intellectual contribution to the work, and approved it for publication.

## Conflict of Interest

The authors declare that the research was conducted in the absence of any commercial or financial relationships that could be construed as a potential conflict of interest.

## Abbreviations

ACE2: angiotensin-converting enzyme 2
ARDS: acute respiratory distress syndrome
CDC: Center of Disease Control and Prevention
COPD: chronic obstructive pulmonary disease
GGOs: ground-glass opacities
COVID-19: coronavirus disease 2019
RBD: receptor-binding domains
CT: computerized tomography
CXR: chest x-ray
FDA: Food and Drugs Administration
HDL: lactate dehydrogenase
HiB: *Haemophilus influenzae* B
IgG: immunoglobulin G
IgM: immunoglobulin M
IL-2: interleukin 2
IL-6: interleukin 6
IL-7: interleukin 7
IL-10: interleukin 10
GCSF: granulocyte colony-stimulating factor
IP-10: interferon-inducible protein
MCP-1: monocyte chemoattractant protein-1
MIS-C: multisystem inflammatory syndrome in children
MIP-1: macrophage inflammatory protein 1 alpha
PICU: pediatric intensive care unit
RNA: ribonucleic acid
RT-PCR: reverse transcription polymerase chain reaction
SARS-CoV-2: severe acute respiratory syndrome coronavirus 2
TMPRSS2: transmembrane protease serin 2
TNF-α: tumor necrosis factor alpha.
